# *Ascaridia galli*, a common nematode in semiscavenging indigenous chickens in Bangladesh: epidemiology, genetic diversity, pathobiology, ex vivo culture, and anthelmintic efficacy

**DOI:** 10.1016/j.psj.2023.103405

**Published:** 2023-12-28

**Authors:** Sumaya Naznin Ritu, Sharmin Shahid Labony, Md. Shahadat Hossain, Md. Haydar Ali, Muhammad Mehedi Hasan, Nusrat Nadia, Akter Shirin, Ausraful Islam, Nusrat Nowrin Shohana, Md. Mahmudul Alam, Anita Rani Dey, Md. Abdul Alim, Anisuzzaman

**Affiliations:** ⁎ Department of Parasitology, Bangladesh Agricultural University, Mymensingh 2202, Bangladesh; † Department of Pathology and Parasitology, Hajee Mohammad Danesh Science and Technology University, Dinajpur 5200, Bangladesh; ‡ Department of Fisheries Technology, Faculty of Fisheries, Bangladesh Agricultural University, Mymensingh 2202, Bangladesh; § Department of Surgery and Obstetrics, Bangladesh Agricultural University, Mymensingh 2202, Bangladesh

**Keywords:** prevalence, epidemiology, anthelmintic efficacy, ascaridiosis

## Abstract

*Ascaridia galli* is the most common nematode in chickens. *Ascaridia galli* is highly prevalent in chickens reared in scavenging or semiscavenging systems. Here, we studied the epidemiology, pathology, genetic diversity, ex vivo culture protocol and anthelmintic sensitivity of *A. galli* prevalent in indigenous chickens in Bangladesh. Through morphological study and molecular analyses, the isolated worms were confirmed as *A. galli*. Of the chickens examined, 45.6% (178 out of 390) were found infected. The male and young chickens were significantly (*P* < 0.05) more prone to *A. galli* infection. Prevalence of the infection was significantly (*P* < 0.05) lower in the summer season. In heavy infections, *A. galli* blocked the small intestine. Marked inflammation, increased mucus production and petechial hemorrhages were evident in the small intestine, particularly in the duodenum. Also, there were desquamation and adhesion of the mucosal villi; degeneration, necrosis of the epithelial cells and goblet cell hyperplasia. The mucosal layer was infiltrated mainly with eosinophils and heterophils. We developed a hen egg white-based long-term ex vivo culture protocol which supported the survival and reproduction of *A. galli* for more than a week. Levamisole (**LEV**) and ivermectin (**IVM**) efficiently killed *A. galli*. However, albendazole (**ABZ**), mebendazole (**MBZ**), and piperazine (**PPZ**) did not kill the worms even at 120 μg/mL and 1mg/mL concentrations, respectively. Taken together, our results suggest that *A. galli* is highly prevalent in semiscavenging chickens in Bangladesh. *Ascaridia galli* can be easily maintained ex vivo in egg white supplemented M199 medium. LEV and IVM, but not ABZ, MBZ and PPZ, can be used for treating and controlling *A. galli* infections in chickens.

## INTRODUCTION

*Ascairdia galli* is a soil transmitted gastrointestinal nematode, which affects chickens and turkeys. The parasite is transmitted through feco-oral route in chickens. Pigeons and other wild birds act as the reservoir hosts of *A. galli*. The prevalence of ascaridiosis ranges between 70 and 80%, especially in indigenous semiscavenging chickens (Mlondo et al., 2022; Shifaw et al., 2023). The life cycle of *A. galli* is direct. Eggs passed with feces embryonate within 2 to 3 wk in the environment with appropriate humidity and temperature (Samour, 2016). Chickens become infected by ingesting the infective eggs containing third larval stage (L3) (Höglund et al., 2023). However, earthworms serve as transport hosts that ingest and accumulate the infective eggs (eggs with L3), and carry the infective stage (Permin et al., 1997). Following ingestion, the infective L3 hatches in the proventriculus or small intestine of chickens. Temperature, carbon dioxide levels, and pH are thought to be the triggering factors that signal the larva to hatch (Samour, 2016). L3 enters into the duodenum and molts to develop L4 for further development. In the lifecycle of *A. galli*, there is no somatic larval migration (Marchiondo et al., 2019) but the larva essentially burrow into the intestinal mucosa and complete the histotrophic stage. In the histotrophic phase, these worms cause the most damage to their hosts. They then re-enter into the small intestine and develop into adults, where they move freely and feed on gut microbes and mucosal tissues (Soulsby, 1982; Shohana et al., 2023). The prepatent period of *A. galli* is 5 to 6 wk (Marchiondo et al., 2019). Some breeds of chickens (e.g., Lohmann Brown) have been shown to have some resistance against *A. galli* (Shohana et al., 2023).

Ascaridiosis affected birds become anemic and suffer from diarrhea. The worm frequently causes hypoglycemia, increased urates, atrophy of the thymus, stunted growth and reduced egg production. In heavy infection, adult parasites can cause intestinal obstructions, which in turn can lead to reduced nutrient absorption and depletion of fat reserves in the liver (Daş et al., 2010; Sharma et al., 2018; Torres et al., 2019; Feyera et al., 2022). Adult worms may also migrate into the oviduct, and become incorporated in eggs (Höglund et al., 2023). Heavy infection is the major cause of atrophy of the thigh and breast muscles, resulting in weight loss. Unthriftiness, drooping of the wings, and bleaching of the head are also seen. Adult worms may even pass with droppings (Soulsby, 1982). Some reports have mentioned that *A. galli* increases the host susceptibility to secondary bacterial infections like *Escherichia coli* and *Pasteurella multocida* (Dahl et al., 2002; Eigaard et al., 2006; Permin et al., 2006).

Vaccines are yet to be developed against *A. galli*, and therefore, control of this worm in poultry mainly depends on prophylactic chemotherapy by anthelmintics. In Bangladesh, mainly piperazine citrate (**PPZ**) is used against *A. galli* infections. However, albendazole (**ABZ**), mebendazole (**MBZ**), levamisole (**LEV**), and ivermectin (**IVM**) are also used to treat *A. galli* infection. But the effectiveness of the above mentioned anthelmintics are questionable. Treatment of specific cases as well as prophylactic mass drug administrations (**MDA**) are recently giving equivocal results that warrant optimization of chemotherapy against *A. galli*. Furthermore, it is essential to develop a standard, long-term (>2 wk) ex vivo culture method for *A. galli*, which will be extremely helpful for new drug discovery, screening of immune sera, understanding the developmental biology and to determine the efficacy of commercially available drugs. Consequently, it will minimize the use of experimental animals/birds, and ensure Replacement, Reduction, and Refinement (**3Rs**) of animal use.

In this study, we have described the present status of *A. galli* infection in semiscavenging chickens along with the species level confirmation of the worm by multiple molecular tools. The risk factors governing the ascaridiosis and pathological changes induced by the worms were also assessed. We also develop an ex vivo culture system for *A. galli* and eventually determined the efficacy of different anthelmintics against *A. galli*. To our knowledge, this is the first report regarding anthelmintic resistance against *A. galli* in Bangladesh.

## MATERIALS AND METHODS

### Study Period and Samples

A cross sectional study was carried out for a period of 5 yr from July, 2018 to June, 2023. A total of 390 chickens of both sexes (male, *n* = 203 and female, *n* = 187) and different ages (young, ≤6 mo, *n* = 209; adult, >6 mo, *n* = 181) were collected and examined. Samples were collected following a sampling calendar throughout the entire study period. To study the temporal distribution, the year was divided into 3 seasons: summer (March–June, *n* = 116), rainy (July–October, *n* = 135) and winter (November–February, *n* = 139). Comparable number of samples were collected in each season and each year.

### Ethical Approval

Live chickens were handled following the guidelines approved by the Animal Welfare and Experimentation Ethics Committee of Bangladesh Agricultural University, Mymensingh (Approval number: AWEEC/BAU/2018 [25]).

### Sample Collection and Postmortem Examination

A total of 390 chickens were collected from the local market of Mymensingh, Bangladesh (coordinate: 24.7460° N, 90.4179° E). Data regarding age, sex, and breed of the chickens were recorded. Chickens were bled properly by cutting carotid arteries following the Muslim slaughtering method and the digestive tract was collected. Each part of the alimentary tract was secured with threads or clamped. Then, the parts of the alimentary tract were separated, and placed individually into separate jars containing phosphate buffered saline (**PBS**). Different parts of the alimentary tract were opened along the long axis using a scissors. After opening, the mucosal surface was gently washed with PBS into a jar. The washings were suspended and the supernatant was discarded. The procedure was repeated several times until the washings became clear.

### Parasite Collection, Preservation, and Tentative Identification

After washing, the nematodes were collected and *A. galli* was identified by preparing temporary slide by adding a drop of lactophenol and following the previously published guidelines (Soulsby, 1982). The worms were then preserved in absolute ethanol in glass vials separately for molecular analysis.

### PCR-Based Confirmation of *A. Galli*

Genomic DNA of male *A. galli* was collected using the QIAamp DNA Mini Kit (Qiagen, Germany). Concentration of DNA was measured by a nanodrop spectrophotometer and PCR was performed. Partial fragments of the mitochondrial gene (*cox1*) were amplified using the previously published primer sets (Malatji et al., 2019). Briefly, PCR amplifications were performed in 25 μL reactions using One Taq Quick-Load 2X Master Mix containing ∼2.5 units of puReTaq DNA polymerase, 10 mM Tris–HCl (pH 9.0), 50 mM KCl, 1.5 mM MgCl_2_, 200 μM of each dNTP and stabilizers including BSA, 10 pmol of each PCR primer (Ag Cox1F: ATT ATT ACT GCT CAT GCT ATT TTG ATG and Ag Cox1R: CAA AAC AAA TGT TGA TAA ATC AAA GG), and 50 ng of genomic DNA. The PCR thermal profile was 15 min at 95°C for initial activation of Taq polymerase, followed by 35 cycles consisting of 30 s at 95°C (denaturation), 40 s at 55°C (annealing), 1 min at 72°C (extension) and a final elongation of 10 min at 72°C (Malatji et al., 2019). PCR product was run on an agarose gel (1.5%) electrophoresis to detect the transcript of the expected size following the methods as described previously (Anisuzzaman et al., 2011).

### Sequencing, Bioinformatic, and Phylogenetic Analysis

PCR products were purified and subjected to sequencing in forward and reverse directions. Sequences were aligned and edited using BioEdit 7.2 software. The obtained sequences have been submitted to the GenBank (OR497510-OR497517). Sequences were compared against the NCBI database using BLASTN (https://blast.ncbi.nlm.nih.gov/Blast.cgi), and sequences with nucleotide identity of approximately 98% were used for further analysis. Sequences were aligned using CLUSTALΩ (https://www.ebi.ac.uk/Tools/msa/clustalo/) software for pair-wise comparisons with previously published sequences (OP114239, OP218593, MT776400, KP982856, MW243594, GU138668, KP982856, GU138669, MW243593, FM178545, KT613889, KT613895, KT613896, KT613902, KT613900, KT613899, LC592822, LC592832, LC592838, LC592821, LC592841, LC592833). Phylogenetic analysis was performed using Neighbor Joining (**NJ**), Maximum likelihood (**ML**) and Maximum parsimony (**MP**) methods, based on the Tamura-Nei model where *cox1* gene of *Haemonchus placei* (KJ724431) was used as an outgroup. Confidence limits were assessed using the bootstrap procedure (1,000 replicates) and other settings were obtained using the default values in MEGA X. A 50% cut-off value was implemented for the consensus tree (Tamura and Nei, 1993).

### Study of Gross and Histopathological Changes

The mucosal surface was carefully examined to detect helminths, especially, nematodes. Mucosal surface around the lodged parasite was examined to detect the pathological changes, if any. Affected parts of the intestine were collected and preserved in 4% paraformaldehyde added with 0.1% glutaraldehyde overnight. It is worth mentioning that chickens were carefully examined before and after slaughtering to detect any acute and/or chronic disease(s). Chickens having only *A. galli* infection were selected for pathological studies and the affected tissues were collected. The fixed tissues were washed extensively with PBS and trimmed off. Taken into a histocassette, the tissue was rinsed with PBS for 20 min. Tissues were then dehydrated through a series of ascending grades of ethanol (50–100%) and cleaned through 2 changes in xylene for 30 min in each. The tissues were then immersed in molten paraffin. Finally, the tissues were placed in stainless steel molds and embedded with molten paraffin to make paraffin blocks which were stored at −20°C until sectioning. Thin sections (5 μm) were made and stained with hematoxylin and eosin stains (**H&E**). Tissues from age- and sex-match noninfected chickens were used as control. At least 3 foci (for control 3 foci and for infected birds 5 foci) from each slide and 3 slides from each tissue were evaluated in a blinded manner. Numbers of infiltrated cells were counted and analyzed.

### Ex Vivo Culture

Collected *A. galli* were washed in sterile PBS supplemented with 200 U/mL penicillin, and 200 μg/mL streptomycin (Sigma-Aldrich, Germany). Adult *A. galli* (2 parasites in 2 mL) were incubated in Medium 199 (M199), DMEM or RPMI 1640 (Sigma-Aldrich) supplemented with fetal calf serum (**FCS**), chicken serum or hen egg white (**hEW**) at different concentrations (5–20%) along with 200 U/mL penicillin, and 200 μg/mL streptomycin (Sigma-Aldrich) in a 6-well flat bottom tissue culture plate (Corning Incorporated). The worms were then incubated at 37°C in 5% CO_2_ in a humidified air for up to 1 wk. For each condition, experiments were performed in triplicates. The medium was replaced every other day and viability parameters of *A. galli* were examined under an inverted microscope (Labomed Inc.). Mortality was evaluated observing motility, pharyngeal pump, and integrity of the cuticle. Worms kept only in the respective media served as a control. Scoring was performed in a blinded manner following the parameters as provided (Supplementary Figure 1).

### Ex Vivo Optimization of Anthelmintics

Technical grade (**TC**; absolutely pure chemical components used for analysis or testing) anthelmintics such as ABZ, MBZ, LEV and IVM were dissolved in DMSO and PPZ in PBS, and stored at −20°C until use. Adult *A. galli* were maintained overnight in M199 supplemented with 20% hEW and incubated overnight in the same condition as mentioned above by adding 200 U/mL penicillin and 200 μg/mL streptomycin. The following morning the media was changed. The selected drugs were then added at different concentrations (1–20 μg/mL for LEV; 0.25–2 μg/mL for IVM; 20–120 μg/mL for ABZ and MBZ, and 50–1,000 μg/mL for PPZ) and were incubated at 37°C in 5% CO_2_ in a humidified air up to 48 h. For each condition, experiments were performed in triplicates. Nontreated *A. galli* were kept as a control. Scoring was performed before (0 h) and at 3, 6, 12, 24, 36 and 48 h post treatment (**p.t**) in a blinded manner.

### Statistical Analysis

The collected data were coded and entered into Microsoft Excel spread sheet. Data obtained in the present study were analyzed through descriptive statistics. For the age-, sex- and season-wise comparison, *Z* test was used. On the other hand, data regarding anthelmintic efficacy were analyzed using 1-way ANOVA followed by Bonferroni post hoc analysis.

## RESULTS

### Overall Prevalence of *A. Galli* Infection and Morphometric Identification

**Figure 1:**
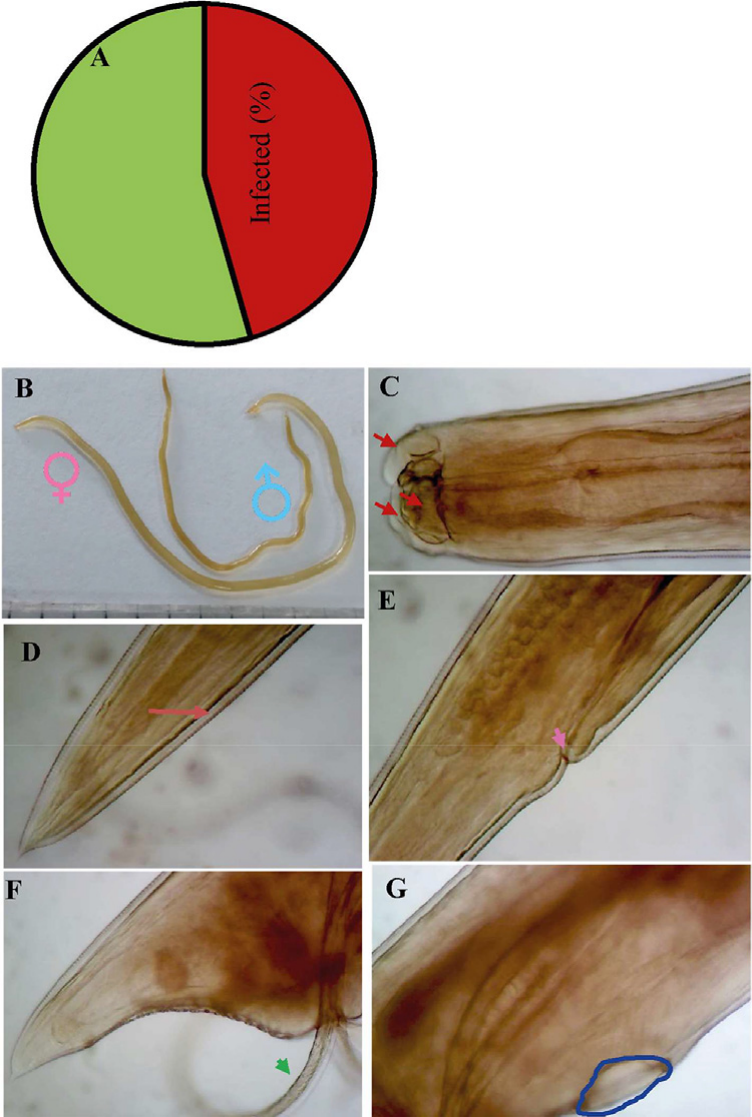
Overall prevalence and morphology of *Ascaridia galli*. A total of 390 chickens of both were collected and examined. Parasites were isolated and identified by morphology and morphometry. (A) Overall prevalence of *A. galli*. (B) Adult male and female *A. galli*. (C) Anterior part of the parasite. (D) Tail of the female parasite. Arrow indicates anus (E) Vulva of the parasite. Arrow indicates vulvar opening. (F) Posterior part of the male. Arrow indicates specule. (G) Precloacal sucker of the male (blue circle).

Of the chickens examined (*n* = 390), 178 (45.6%) chickens were found infected with *A. galli* (Figure 1A). Both adult *A. galli* and larval stages of the parasites were detected. Parasites were detected mostly in the duodenum and also in the jejunum. The body of *A. galli* was semitransparent, creamy-white, and cylindrical. The females (65–115 mm) were longer than the males (45–81 mm) (Figure 1B). The anterior end was characterized by a prominent mouth, which was surrounded by 3 large, trilobed lips with tooth-like denticles (Figure 1C). The cuticle was transversely striated and the cuticular alae were poorly developed. The tail in females was blunt and straight (Figure 1D, E) but in males it was shorter, pointed and curved. However, in males, 10 pairs of caudal papillae and a precloacal sucker were found in the tail (Figure 1F, G), conforming to the characteristic features of *A. galli* as described previously (Soulsby, 1982).

### PCR- and Sequencing-Based Confirmation of Species of *A. Galli*

We conducted PCR using species specific primers, which produced an amplicon approximately 530 bp in length (Supplementary Figure 2), confirming the parasite as *A. galli*. To further confirm the species of the worm, the PCR products were sequenced and the data were aligned, edited and analyzed. Identical sequences and the level of homology of the newly obtained sequences were searched. The study revealed that the new sequences (OR497510-OR497517) had >99 to 100% identity with the previously deposited reference *cox 1* (OP114239) sequence of *A. galli*.

### Phylogenetic Analysis of the Present Isolates With the Others

**Figure 2:**
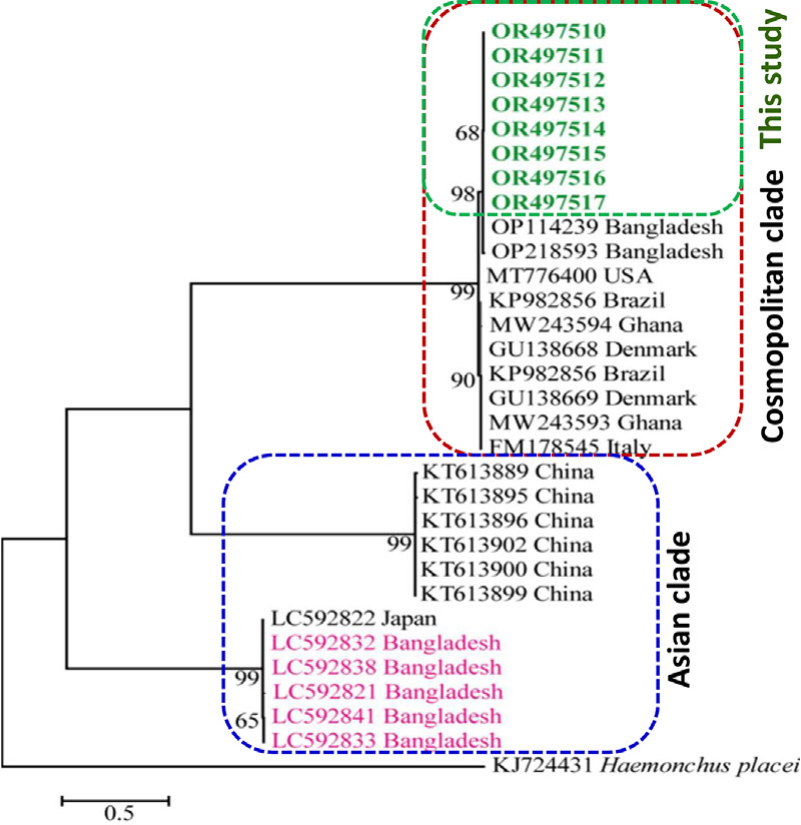
*Cox 1*-based phylogram of *Ascaridia galli*. Genomic DNA of male *A. galli* was collected and PCR was performed. PCR products were purified and sequenced. Phylogenetic analysis was performed based on the Tamura-Nei model where *cox1* gene of *Haemonchus placei* was used as an outgroup. Confidence limits were assessed using the bootstrap procedure (1,000 replicates).

To determine the phylogenetic relationship of the isolates trees were constructed using *cox 1* sequences obtained in the present study as well as those of *A. galli* previously submitted to GenBank. We constructed trees using NJ, ML, and MP methods, which gave almost the same results. Therefore, for the convenience of presenting results and to avoid redundancy, we described only the ML tree. By the phylogenetic analysis, it was revealed that the newly obtained sequences of the present study formed a unique cluster with previously deposited *cox1* sequences of *A. galli* from different countries of Europe, America, Africa and with few sequences reported from Bangladesh as well. Our analysis showed that there were mainly 2 clades of *A. galli*, of which one is prevalent in China, Japan and Bangladesh (deposited from previous study to the GenBank under the accession number: LC592821, LC592832, LC592833, LC592838, LC592841), therefore, we named it as an Asian clade. However, another clade is cosmopolitan, which is distributed in the major continents of the world covering Asia, Africa, Europe, and America. Isolates of the present study formed a cluster with the cosmopolitan clade supported by the strong bootstrap values (Figure 2). The phylogenetic analysis suggests that both the Asian and cosmopolitan clades are prevalent in Bangladesh.

### Common Factors Involved in Ascaridiosis of Chickens

**Figure 3:**
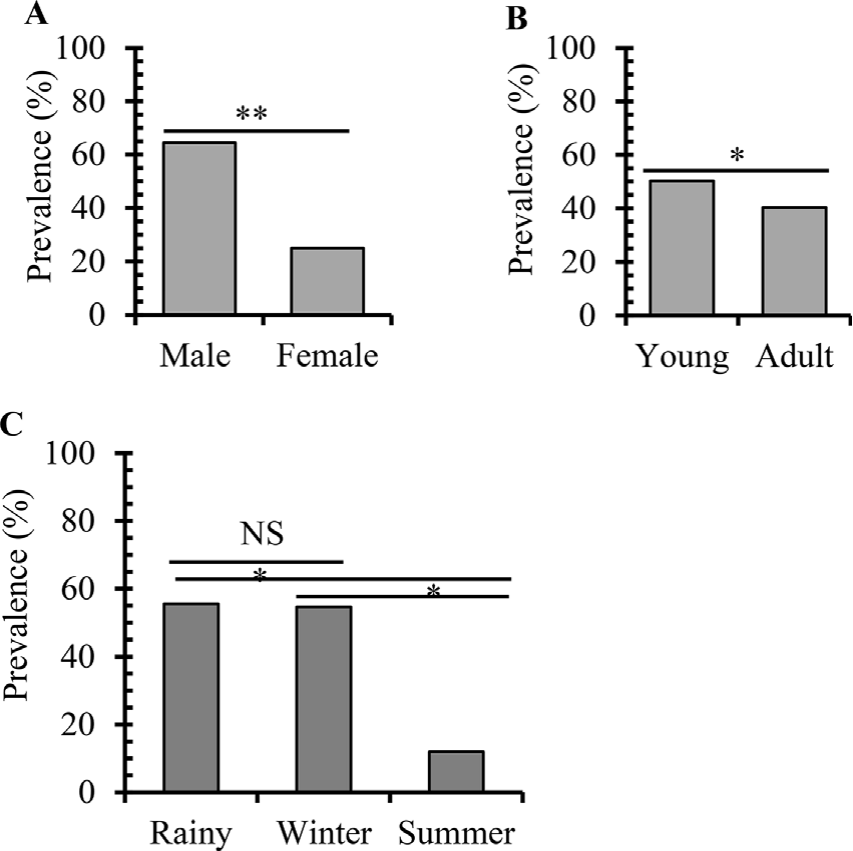
Factors associated with the Ascaridia galli infections. A total of 390 chickens of both sexes (male, *n* = 203 and female, *n* = 187) and different ages (young, ≤6 mo, *n* = 209; adult, >6 mo, *n* = 181) covering different seasons such as summer (March-June, *n* = 116), rainy (July-October, *n* = 135) and winter (November-February, *n* = 139) were collected and examined. (A) Sex-wise infection of the worm. (B) Age-wise infection of *A. galli* in chickens. (C) Prevalence of the worm in different seasons. **P < 0.01; *P < 0.05.

In this study, we observed that prevalence of the worms was significantly (*P* < 0.05) higher in the male birds (64.5%, 131 out of 203) than in the females (25.1%, 47 out of 187) (Figure 3A). To determine the impact of age, chickens were divided into 2 age groups such as young (<6 mo of age) and adult (≥6 mo of age). Accordingly, a comparable number of chickens (209 young and 181 adult chickens) were examined. The study revealed that young birds (50.2%, 105 out of 209) were more susceptible to *A. galli* infection than the adults (40.3%, 73 out of 181) (Figure 3B).

We also examined a comparable number of samples in 3 distinct seasons such as summer (March–June), rainy (July–October) and winter (November–February) in Bangladesh (Labony et al., 2020). The prevalence of the infection was almost the same in the rainy season (55.6%, 75 out of 135) and winter season (54.7%, 76 out of 139). However, it was significantly (*P* < 0.05) lower in the summer (23.3%, 27 out of 116) season (Figure 3C).

### Pathology of *A. Galli* Infection in Indigenous Chickens

**Figure 4:**
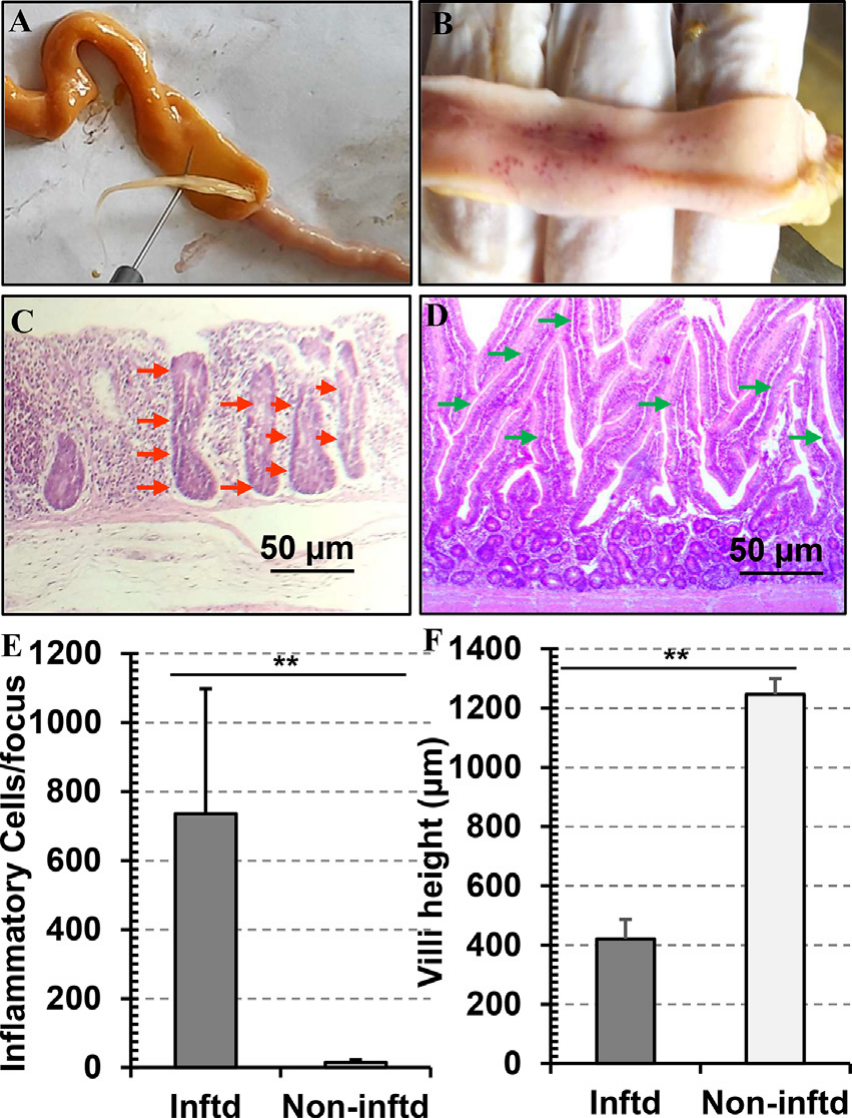
Gross and histopathological changes induced by the worm. Birds were euthanized and different parts of intestines were opened and examined. (A) Blockage of intestinal lumen by *A. galli*. (B) Petechial hemorrhages detected at the site of lodgment of *A. galli*. (C) Histopathological changes detected in the intestinal tissues collected from intestine of infected chickens. (D) Histological pattern of intestine of noninfected chickens. (E) Count of inflammatory cells infiltrated in the intestinal tissues of the infected chickens. (F) Pattern of inflammatory cells in the noninfected chickens (control). Data were presented as mean ± SE. **P < 0.01.

In heavy infections, blockage of small intestine, petechial hemorrhage, marked inflammation and increased mucus production were commonly found in the duodenum. Hemorrhagic enteritis and/or fibrinous enteritis along with clotted blood and necrotic patches were also observed (Figure 4A and B). *Ascaridia galli* and lesions were also detected in the other parts of the small intestine, however, we did not find any worm in the large intestine. Histopathological examination of the sections of duodenum revealed desquamation and adhesion of the mucosal villi, degeneration and necrosis of the mucosa. Goblet cell hyperplasia was commonly evident. The mucosal layer was infiltrated mainly with eosinophils and heterophils as well (Figure 4C). Such changes were not seen in the duodenal sections of age- and sex- matched noninfected control chickens (Figure 4D). Quantitative histological analysis revealed that there was massive infiltration of inflammatory cells (735 ± 362.7 cell/focus) in the affected parts of the intestine, particularly where a bundle of adult *A. galli* were found. In contrast, very few cells were detected in the intestine of the control chickens (15 ± 7.6 cell/focus) (Figure 4E). Our experiment revealed that height of the villi of the affected intestinal part was reduced markedly (421 ± 65.67 μm) compared to that of control chickens (1,246.5 ± 53.17 μm). Villi of the affected intestinal parts were found denuded and fused together. In contrast, in the control chickens, intestinal villi remained elongated with free borders and tips having fulminating appearance (Figure 4F).

### A Long-Term Ex Vivo Culture Protocol Was Developed

**Figure 5:**
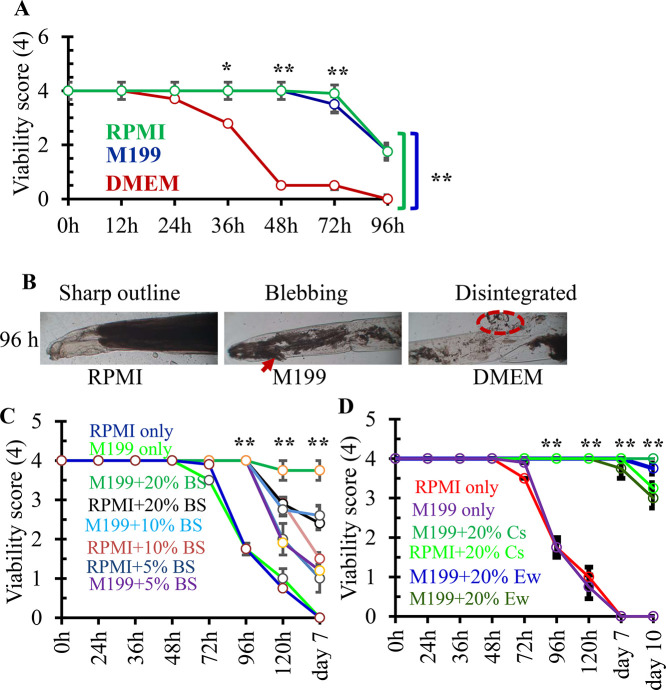
Development of a suitable, long-term and cost-effective culture protocol for *Ascaridia galli*. (A) Viability of the worm in different media. Collected *A. galli* were washed and incubated in Medium 199 (M199), DMEM or RPMI 1640 supplemented with or without fetal calf serum (FCS), chicken serum or hen egg white (hEW) at different concentration (5–20%) in a 6-well flat bottom tissue culture plate at 37°C in 5% CO2 in a humidified air at indicated time points. (A) Survival of the worm in different media. (B) Different changes induced is dead or devitalized worms. (C) Viability of the worm in bovine serum. (D) Viability of the worm in chicken serum and hen egg white. BS, bovine serum; Cs, chicken serum; hEW, hen egg white. Data were presented as mean ± SE. **P < 0.01; *P < 0.05.

For the precise study of biology, drug screening and response to immune sera, a long-term ex vivo culture protocol of *A. galli* is essential. To develop this, we incubated adult *A. galli* in different commercially available media such as RPMI, M199 and DMEM up to 7 d and examined at different time points. To determine the viability score, we observed several points of the worms such as motility, outline, pharyngeal pump and lip movement (Supplementary Figure 1). On the basis of collective scoring, the present study revealed that DMEM media failed to provide sufficient support for the survival of the worm for more than 48 h. In this media, viability of the parasite dropped rapidly within 48 h and reached to 0.5 (out of 4). All parasites died by 96 h. On the other hand, RPMI and M199 efficiently supported the survival and reproduction of the worms, and the viability score remained >3.5 even at 72 h of incubation then gradually declined to ∼2 by 96 h (Figure 5A and B); thus, we decided to use either RPMI or M199 for the establishment of the ex vivo culture protocol.

### Development of a Cost Effective and Easy Long-Term Culture Protocol for *A. Galli*

Since RPMI or M199 provided the highest support for viability, therefore, we tested the impact of sera of different avian and nonavian species on the viability of the worms using these 2 media. We found that addition of bovine sera (20%) significantly increased the viability of the worm and *A. galli* survived up to 7 d with an average viability score of 3.75 in M199 media, however, at that time point viability score of the worm in 20% bovine sera supplemented RPMI was only 2.4 (Figure 5C and D). On the other hand, 20% chicken sera enriched M199 provided support to the worm up to 10 d of incubation with a viability of 4.0, suggesting 20% chicken serum added M199 can be a suitable ex vivo platform to culture adult *A. galli* for extended period of time. Since adult *A. galli* can also survive in chicken eggs, we also tested the impact of hEW on the viability of the worm. Surprisingly, 20% hEW supplemented M199 supported the survival of the worm up to 10 d with a viability score of 3.75. Since hEW is very cheap and also provided a comparable viability when compared with chicken serum (Figure 5D), we used 20% egg white enriched M199 as the ex vivo culturing platform for adult *A. galli* and for determining the efficacy of anthelmintics.

### Determination of Efficacy of Commercially Available Anthelmintics

**Figure 6:**
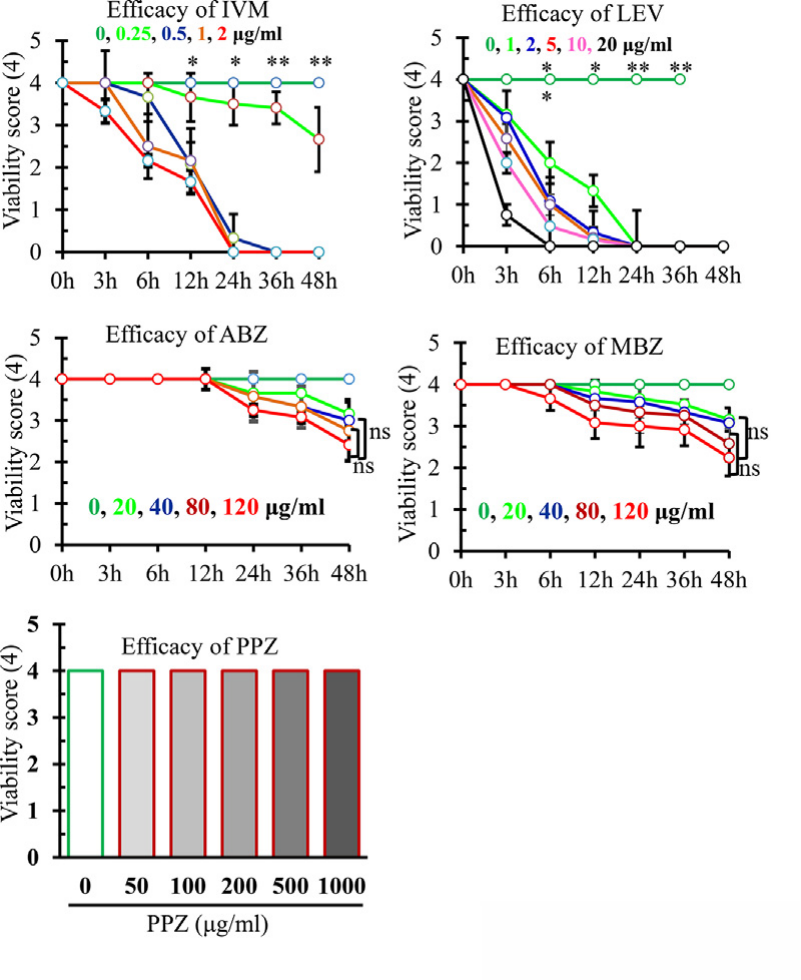
Efficacy of different anthelmintics against *Ascaridia galli*. Adult *A. galli* was maintained overnight in M199 supplemented with 20% hEW and 200 U/mL penicillin and 200 μg/mL streptomycin. In the following morning, the medium was changed and drugs were added at different concentrations (1–20 μg/mL for LEV; 0.25–2 μg/mL for IVM; 20–120 μg/mL for ABZ and MBZ, and 50–1,000 μg/mL for PPZ), and were incubated at 37°C in 5% CO2 in a humidified air up to 48 h. *Ascaridia galli* kept in media alone served as a control. Data were presented as mean ± SE. ABZ, albendazole; MBZ, mebendazole. LEV, levamisole; IVM, ivermectin. **P < 0.01; *P < 0.05.

Following hEW-based (M199 supplemented with hEW) long-term ex vivo culture protocol, we tested the efficacy of several commercially available anthelmintics. The results revealed that IVM and LEV efficiently reduced the viability score of the worm in a concentration dependent manner. At 2 μg/mL concentration, IVM markedly reduced the viability score (1.66 ± 0.28) within 12 h and killed all *A. galli* at 24 h of post treatment (p.t). We also found LEV as an effective anthelmintic, which at the same concentration (2 μg/mL) dramatically reduced the viability score (0.33 ± 0.52) of the treated worms within 12 h and killed all *A. galli* at 24 h p.t. Since, the therapeutic dose of LEV (8–10 mg/kg) is much higher than that of IVM (0.2–0.4 mg/kg), therefore, we also tested the efficacy of LEV at its higher concentration (20 μg/mL) against *A. galli*. During our study, we found that the viability score of the worms drastically dropped to <1 (viability score, out of 4) within 3 h of treatment at the foresaid concentration. And, LEV at 20 μg/mL concentration killed all worms by 6 h, suggesting its very potent efficacy against *A. galli*. However, ABZ and MBZ did not kill *A. galli* even at 120 μg/mL within 48 h p.t., indicating that ABZ and MBZ are not effective enough against the prevalent *A. galli* infection in Bangladesh. Also, PPZ at the concentration of 1 mg/mL did not kill *A. galli* (Figure 6). Taken together, our results suggest that only LEV and IVM were highly effective in killing *A. galli* prevalent in Bangladesh.

## DISCUSSION

Chickens make up 90% of the world poultry meat production, followed by turkeys with 5%, ducks with 4% and geese and guinea fowl with 1% (Food and Agriculture Organization of the United Nations, 2022). Chickens are the most popular among the poultry as a source of meat and eggs in developing countries like Bangladesh. Although *A. galli* infections in chickens has been reported, however, epidemiological factors associated with the infection and anthelmintic efficacy against the worm have not been studied in Bangladesh. In addition, an ex vivo long-term culture protocol has not yet been established. In the present study, we have provided the updated information regarding the present status of *A. galli* infection with its epidemiology, pathology, genetic analyses, development of an easy, cost-effective, long-term ex vivo culture protocol and the efficacy of commercially available common anthelmintics against *A. galli* ex vivo.

The present study still showed very high prevalence (45.6%) of ascaridiosis in indigenous chickens reared in semiscavenging system in Bangladesh. However, about a decade ago, a study reported 87.5% prevalence of *A. galli* infection in indigenous chickens in Bangladesh (Rabbi et al., 2006). Another cross-sectional study in Bangladesh, reported 70 to 85% prevalence of ascaridiosis in chickens (Ferdushy et al., 2016). *A. galli* has a global distribution, covering almost all climatic zones. However, the prevalence of ascaridiosis in indigenous chickens was reported comparatively lower in India (32.97%) (Ara et al., 2021) and Pakistan (24.5%) (Hafiz et al., 2015) as compared to our current study. Due to rapid expansion of free-range chicken rearing in conventional and organic farms, the occurrence of *A. galli* has also increased in some European countries like Germany (88%) (Kaufmann et al., 2011) and Sweden (77.1%) (Jansson et al., 2010). The prevalence of ascaridiosis was also reported in indigenous chickens from African countries like Tanzania (32.3%) (Permin et al., 1997) and South Africa (22.2–43.8%) (Mukaratirwa and Khumalo, 2010). The variation in the prevalence rate of chicken ascaridiosis in different studies may be due to difference in the geographical location of the research areas, methods of detection, sample size and age of the chickens. In Bangladesh, the decreasing trends of the prevalence of the worm may be due to the increased facilities of the veterinary services, awareness of the people and the gradual motivation of people regarding harmful effects of the helminths as well as the positive changes of their attitude and practice of regular deworming.

The worm can be identified up to the genus level through morphological and morphometrical analyses, however, confirmation of the species requires the help of multiple molecular tools. In this study, we utilized a *cox1*-based species-specific primer for the confirmation of the species as *A. galli*. Through PCR, we obtained an amplicon of 530 bps, which was also observed in other studies (Katakam et al., 2010). To validate the PCR, we sequenced the products and through bio-informatic analysis we found 32 *cox 1* sequences that were almost identical to the sequences we obtained, ascertaining the species of the worm as *A. galli*. Phylogenetic analysis revealed 2 clades such as 1) Asian clade and 2) Cosmopolitan clade. Our study showed that the cosmopolitan clade is composed of *A. galli* sequences from USA, Brazil, Ghana, Denmark, Italy and Bangladesh. On the other hand, the Asian clade represents sequences from China, Japan and also in Bangladesh (sequences from previous studies) (Liu et al., 2016; Biswas et al., 2021; Zhao et al., 2022). *Asciridia galli* can infect wild birds as well (Shohana et al., 2023), therefore, intercontinental distribution of the same clade is not surprising. However, along with the data obtained from the previous studies, the present study unambiguously proved that both genotypes are circulating in Bangladesh. Prevalence of multiple genotypes in a country may lead to the variation in susceptibility, severity of the infection and development of drug resistance through interclade fertilization.

Sex-related prevalence of *A. galli* in indigenous chickens showed significantly (*P* < 0.05) higher infection in the males, which is in agreement with the previous findings (Sarba et al., 2019). It is difficult to explain why male birds were more likely to be infected with the worm. However, it has been reported that male sex hormones make the male individuals more susceptible to parasitic infections (Klein, 2004; Sarba et al., 2019). One controlled experiment targeting the estimation of effect of gonadal hormones on the establishment of *A. galli* infections in chickens showed that injections of α-estradiol benzoate caused a temporary retardation in the mean rate of growth of the *A. galli* but injections of testosterone propionate caused a temporary accelerate the growth of the worm (Sadun, 1951). In addition, male sex hormones greatly enhance the roaming behavior of the male (Klukowski and Nelson, 2001), which may expose them to more infective stage. Furthermore, testosterone has been reported to cause immunosuppression (Roberts et al., 2004); thus, may facilitate the establishment, survival and propagation of the worm in male hosts.

Of the age groups, prevalence of infection was found to be higher in younger chickens of ≤6 mo of age as compared to the adults. One research group also found higher prevalence of *A. galli* infection in young chickens (Sarba et al., 2019). It has been observed that chickens older than 3 mo of age develop nonspecific immunity against *A. galli* due to age-dependent proliferation of goblet cells in the gut of chickens. Since the number of goblet cells increases with the advancement of age in chickens, the mucus production also increases in the gut lumen and the excessive mucus hampers the establishment of newly hatched larvae in the gut wall. Furthermore, in young chickens (<3 mo of age), the worm matures rapidly and the histotrophic phase of *A. galli* in young chickens is also shorter (Permin et al., 1997; Shohana et al., 2023). In addition, the older host is able to mount an immune response to the penetrating larvae, and the larvae do not develop into adults, but hide in the mucosa of the small intestine (Brar et al., 2016; Höglund et al., 2023) . From this study and the knowledge generated from other research (Shohana et al., 2023), it is clear that young birds are more likely to be infected with *A. galli*, therefore, young birds particularly ≤3 mo of age should be reared separately.

In this study, the prevalence of ascaridiosis in chickens varied according to the seasons. We found almost equal prevalence of *A. galli* infection in the rainy and winter seasons and these were significantly higher than that in the summer. Different studies in different ecological zones and experimental settings reported comparable seasonal dynamics of ascaridiosis in chickens. A recent study reported that the prevalence for *Heterakis gallinarum* and *A. galli* was comparatively higher during the hot-wet season than the cold-dry season (Makalo et al., 2022). In fact, desiccation is very detrimental for the survival and development of eggs of ascarids, including *A. galli* (Tarbiat et al., 2015). Therefore, eggs deposited in the late winter and in the summer are less likely to develop in the environment; thus, the lowest prevalence of *A. galli* in the summer is not surprising. In addition, earthworms act as the transport host of *A. galli*, which remain abundant from the late summer to the end of the rainy season in Bangladesh, and the chances of getting infection become very high during this period. But in the dry summer earthworms undergo hibernation and freely moving earthworms are very rarely seen on the surface, which may contribute to the low infection in the summer (Saha et al., 2009).

This study observed variable degrees of pathological lesions due to ascaridiosis in chickens, which varied from catarrhal inflammation to hemorrhagic enteritis. Similar types of lesions were observed in a previous study (Rabbi et al., 2006). In some cases, we found necrotic plaque which is supported by 2 previous observations (Permin et al., 1997; Ferdushy et al., 2016) . The degree of severity largely depends on the load of *A. galli*. Heavy infections resulted in blockage of small intestine, petechial hemorrhage in the duodenum, marked inflammation and increased mucous secretion. Usually, *A. galli* lives on gut microbiota and mucosal tissues. Gut tissue feeding activity may lead to the development of enteritis and hemorrhages. Also, *A. galli* frequently moves inside the intestine, therefore, a single worm can induce injuries at multiple sites (Soulsby, 1982; Shohana et al., 2023). In addition, adult worms, particularly in heavy infections, exert pressure on the gut wall, resulting desquamation and denudation of villi, and eventually ulceration in the intestinal mucosa. Immature worms have a well-defined histotrophic phase, in which the L3 stage penetrates the crypt of the intestinal glands and stay there for a variable time. The duration of the histotrophic phase is dose dependent and may persist up to 54 d (Soulsby, 1982). During this phase, larvae can cause extensive damage in the glandular epithelium, even lead to hemorrhage in the mucosa. Moreover, the embedded larvae may elicit hyperplasia of mucus secreting goblet cells, resulting in excessive secretion of mucus. In long standing cases, necrotic patches and reddish spots on the intestinal wall can develop. Also, during histotrophic phase, the penetrating larvae can cause petechial hemorrhages in the intestinal mucosa (Shohana et al., 2023).

The present study showed that *A. galli* can survive well and lay eggs both in chicken serum and hEW supplemented M199 but bovine serum failed to provide the support to the same extent. The worm mainly causes infection in chickens and lives in the small intestine, particularly, in the duodenum (Soulsby, 1982). *Ascaridia galli* has never been reported from any mammals. Therefore, rapid decrease of the viability score of the worm in presence of bovine serum is not surprising. In our previous studies on *Schistosoma mansoni,* we showed that serum isolated from unnatural hosts prevent survival and development of the parasites (Frahm et al., 2019). Through in-depth analysis using specific gene depleted mice, we showed that soluble serum factors, other than complements and nonspecific immunoglobulins (**Igs**) provide a band of protection in the establishment of a particular parasite in an unnatural host; thus, govern the host specificity (Anisuzzaman et al., 2021).

Live adult *A. galli* have been detected in eggs (Jabalera et al., 2022; Wang and Wang, 2022). Therefore, long-term survival of *A. galli* in hEW supplemented culture media is not surprising. Although chicken serum provides better support for the survival of the parasite but it is expensive. In contrast, collection of egg white is easier and cheaper. Moreover, hEW contains lysozyme, which has antibacterial activity and is used as a nonspecific antibacterial agent even in human medicine (Chen et al., 2022; Bergamo and Sava, 2023). Therefore, addition of egg white in the culture milieu provides a rich source of nutrients and possibly prevents bacterial growth as well.

Through several trials using adult worms from different batches of chickens, we found that PPZ did not work well against *A. galli* even at 1 mg/mL concentration, suggesting resistance of *A. galli* to this anthelmintic. In the early 20th century, PPZ had been marketed and recommended for treating ascaridiosis. Since then, the drug had been used as the most common anthelmintic against *A. galli*. In Bangladesh, anthelmintics are commonly prescribed without coprological assessment. This indiscriminate use of the anthelmintic for a long time along with under dosing may contribute to the development of anthelmintic resistance (Mitra et al., 2023). In addition to PPZ, efficacy of ABZ and MBZ is also questionable. ABZ and MBZ are frequently used benzimidazole (**BMZs**) anthelmintics. Since their discovery, these drugs had been used against different types of helminths. Due to their high safety index and broad spectrum activity, these anthelmintics are prescribed against different species of nematodes, and also against tapeworms and flukes. In general, BMZs have higher affinity to the β-tubalins of worms than that of mammals. BMZs inhibit polymerization of β-tubalins; thus, prevent their assembly into microtubules. Therefore, they induce degenerative changes in the gut cells of the worms exposed. Due to low absorption and bio-availability, relatively higher dose (10 mg/kg body weight) of these drugs are required. However, therapeutic dose of ABZ and MBZ is almost comparable to that of LEV (8–10 mg/kg) (Brander et al., 1982). In our ex vivo experimental settings, LEV killed all worms even at 2 μg/mL concentration within 24 h but ABZ and MBZ did not kill the worms at even 120 μg/mL despite of extension of incubation time (48 h). Thus, we speculate that *A. galli* prevalent in Bangladesh may develop resistance to ABZ and MBZ. Although ABZ- and MBZ-resistance to *A. galli* is yet to be determined but benzimidazole resistance to *Haemonchus contortus*, a blood feeding stomach worm of ruminants have been reported (Dey et al., 2020; Parvin et al., 2022; Parvin et al., 2023). On the other hand, in this study, LEV and IVM have been found to be very effective in killing adult *A. galli*. LEV and IVM had been shown to kill several nematodes both in ex vivo and in vivo researches (Dey et al., 2020; Parvin et al., 2023), including *A. galli* in the present studies. *Ascaridia galli* is the most economically important nematode of poultry and prevalent in the major parts of the world (Ara et al., 2021; Shohana et al., 2023). However, control of the worm mainly depends on anthelmintic therapy (Daş et al., 2010; Torres et al., 2019; Shohana et al., 2023). Therefore, findings of the present study, particularly information regarding anthelmintic efficacy will help to minimize the economic losses caused by *A. galli* globally.

## CONCLUSIONS

Ascaridiosis is still one of the most prevalent nematode infections in the indigenous chickens in Bangladesh. PCR and sequence analysis confirmed the species level identification of the worm as *A. galli.* Age, sex and seasons of the year greatly influenced the prevalence of the worm. The worm induced various gross and histological alterations on the mucosal layer of the intestine of the chickens. Chicken serum or hEW supplemented M199 were shown to be effective in supporting the viability of *A. galli* in ex vivo culture. LEV and IVM were found to be highly effective against adult *A. galli* but PPZ, although still widely used in poultry targeting *A. galli*, was not effective. The study provides data, which will be helpful in establishing protocols to control and clinical management of *A. galli*.

## Appendix Supplementary materials

Supplementary Figure 1. Culture and scoring of *Ascaridia galli* (Created with BioRender.com). Image, application 1

Supplementary Figure 2. PCR band showing amplicon size. *Cox 1* gene of *Ascaridia galli* was amplified and PCR products were separated in an agarose gel. M, molecular weight marker; S1 to S6, gDNA isolated from 6 different male *A. galli*; N, negative control. Image, application 2

## Footnotes

Supplementary material associated with this article can be found in the online version at doi:10.1016/j.psj.2023.103405.
