# Supplementary figures and images for: Ascaridia galli, a common nematode in semiscavenging indigenous chickens in Bangladesh: epidemiology, genetic diversity, pathobiology, ex vivo culture, and anthelmintic efficacy
Source: Poult Sci. 2023 Dec 28;103(3):103405. doi: 10.1016/j.psj.2023.103405 (PMC10809094; doi:10.1016/j.psj.2023.103405)

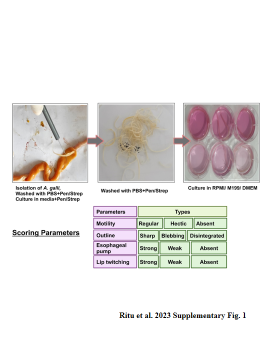

Supplement: Supplementary file 1 — Supplementary Figure 1. Culture and scoring of Ascaridia galli (Created with BioRender.com). [file mmc1.docx]

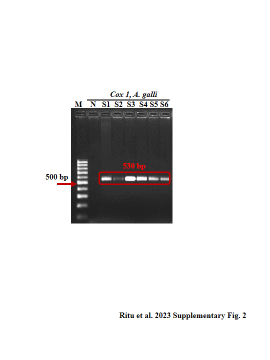

Supplement: Supplementary file 2 — Supplementary Figure 2. PCR band showing amplicon size. Cox 1 gene of Ascaridia galli was amplified and PCR products were separated in an agarose gel. M, molecular weight marker; S1 to S6, gDNA isolated from 6 different male A. galli; N, negative control. [file mmc2.docx]
